# Screening and oenological property analysis of ethanol-tolerant non-*Saccharomyces* yeasts isolated from *Rosa roxburghii* Tratt

**DOI:** 10.3389/fmicb.2023.1202440

**Published:** 2023-06-01

**Authors:** Yinfeng Li, Peipei Ding, Xiaoyu Tang, Wenli Zhu, Mingzheng Huang, Mei Kang, Xiaozhu Liu

**Affiliations:** ^1^Guizhou Institute of Technology, Guiyang, China; ^2^Key Laboratory of Microbial Resources Collection and Preservation, Ministry of Agriculture and Rural Affairs, Beijing, China

**Keywords:** ethanol tolerance, non-*Saccharomyces* yeast, *Rosa roxburghii* Tratt, fruit wine, volatile aroma

## Abstract

Ethanol tolerance is crucial for the oenological yeasts. *Rosa roxburghii* Tratt, a Rosaceae plant native to China, is rich in nutritional and medicinal ingredients. In this study, ethanol-tolerant non-*Saccharomyces* yeasts were screened, and their oenological properties were further evaluated. Three ethanol-tolerant yeast strains (designated as C6, F112, and F15), which could tolerate 12% (v/v) ethanol treatment, were isolated from *R. roxburghii*, and identified as *Candida tropicalis*, *Pichia guilliermondii*, and *Wickerhamomyces anomalus*, respectively. The winemaking condition tolerances of these ethanol-tolerant yeast strains were similar to those of *Saccharomyces cerevisiae* X16. However, their growth, sugar metabolic performance and sulphureted hydrogen activities, were different. The β-glucosidase production ability of strain *W. anomalus* F15 was lower than that of *S. cerevisiae* X16, and strains of *C. tropicalis* C6 and *P. guilliermondii* F112 were similar to *S. cerevisiae* X16. Electronic sensory properties of the *R. roxburghii* wines fermented using ethanol-tolerant yeasts together with *S. cerevisiae* showed no significant differences. However, the mixed inoculation of the ethanol-tolerant yeast strains with *S. cerevisiae* could regulate the volatile aroma characteristics of the fermented *R. roxburghii* wine, enriching and enhancing the aroma flavor. Therefore, the selected ethanol-tolerant yeasts have the potential for application in the production of unique *R. roxburghii* wine.

## Introduction

The flavor characteristics and quality of fruit wine are determined by various factors, including the type of fruit, the brewing process, and the metabolic activity of the selected yeast (Wei et al., 2019). Yeast can be classified into two categories based on their fermentation characteristics and physiological properties: *Saccharomyces cerevisiae* and non*-Saccharomyces* yeasts (Jolly et al., 2014). *S. cerevisiae* is preferred for its high fermentation activity and strong ethanol tolerance, making it a popular choice for fruit wine production, and it is readily available for purchase by producers (Parapouli et al., 2020). However, the commercial varieties of wine yeast are limited, leading to high similarity in the flavor characteristics of fermented fruit wines and a lack of complexity in taste and flavor. As a result, product homogenization is common, which does not meet the diverse needs of consumers for product diversity. Non*-Saccharomyces yeast* refers to a diverse group of yeast species that also play a crucial role in winemaking. This group includes *Hanseniaspora uvarum* (Pietrafesa et al., 2020), *Wickerhamomyces anomalus* (Padilla et al., 2018), *Candida tropicalis* (Egue et al., 2018), etc.

Research has shown that non*-Saccharomyces* yeast can metabolize a greater variety of compounds during fruit wine fermentation, resulting in more complex and aromatic wine characteristics that enhance the overall flavor quality (Morata et al., 2019). However, non*-Saccharomyces* yeast is typically more sensitive to ethanol, which accumulates during the fermentation process and can inhibit its growth and induce cell death, ultimately reducing fermentation efficiency (Contreras et al., 2014). Therefore, the screening of non*-Saccharomyces* yeast strains with higher ethanol tolerance is of great practical significance for the production of distinctive fruit wines.

*Rosa roxburghii* Tratt, a perennial plant belonging to the Rosaceae family and the *Rosa* genus, is widely distributed in southwestern China, such as Guizhou, Sichuan, and Yunnan (Liu X. et al., 2020). The fruit of *R. roxburghii* is rich in nutrients, such as vitamin C, polysaccharides, and carotenoids (Liu et al., 2021a). Moreover, it contains abundant bioactive substances, such as flavonoids, superoxide dismutase (SOD), and organic acids, which give it good medicinal value (Wang et al., 2021). However, due to its high content of phenolic and acidic compounds, the fresh fruit tastes sour and astringent in taste, making it unsuitable for consumption. Therefore, fermenting the fruit into *R. roxburghii* fruit wine is more appropriate (Liu et al., 2021b). Currently, the yeast strains used in *R. roxburghii* fruit wine production mostly come from the active dry yeast used in grape wine production rather than from the indigenous yeast strains of *R. roxburghii*. This leads to poor adaptability of the strains and serious homogenization of the resulting *R. roxburghii* wine. Therefore, screening and isolating excellent indigenous yeast strains of *R. roxburghii* with perfect brewing characteristics, especially non*-Saccharomyce*s yeasts, will promote the healthy development of *R. roxburghii* fruit wine.

In our preliminary research, we used high-throughput sequencing technology to identify the diversity and population changes of non*-Saccharomyces* yeasts during the spontaneous fermentation process of *R. roxburghii* fruit (Liu X. Z. et al., 2020). Additionally, we isolated 80 cultivable non*-Saccharomyces* yeasts from the spontaneous fermentation broth of *R. roxburghii* fruit using culture-dependent approach (Liu X. Z. et al., 2020). In this study, ethanol-tolerant strains were screened from our previously isolated culturable non*-Saccharomyces* yeasts, and then species of these ethanol-tolerant yeasts were identified based on morphology and molecular approaches. In addition, we also analyzed brewing characteristics of these ethanol-tolerant yeasts. Moreover, aroma and quality characteristics of *R. roxburghii* fruit wines were further evaluated by co-inoculation of these non*-Saccharomyces* yeasts together with *S. cerevisiae* as fermentation starter. The results obtained from the present study were helpful to explore potential high-quality brewing strains for the production of characteristic *R. roxburghii* fruit wine.

## Materials and methods

### Yeast strains

The reference strain used in this study was the commercial *S. cerevisiae* X16 obtained from Laffort Company (France). A total of 80 strains of non-*Saccharomyces* yeasts, isolated from spontaneous fermentation of *R. roxburghii* were screened for ethanol-tolerant strains. All yeasts cells were cultured on yeast extract peptone dextrose (YEPD) solid medium (1% yeast extract, 2% peptone, 2% glucose, and 2% agar) containing 100 mg/L of Chloramphenicol at 28°C for 72 h and then stored at 4°C for later use.

### Screening and of identification of ethanol-tolerant non-*Saccharomyces* yeast strains

Ethanol-tolerant strains were screened by culturing them in YEPD broth (1% yeast extract, 2% peptone, and 2% glucose) containing 12% (v/v) ethanol with the initial concentration of 10^8^ cfu/ml, and the yeast cells were cultured at 28°C with shaking at 180 rpm for 36 h. The optical density (OD) values were measured at a wavelength of 600 nm using a spectrophotometer (Hitachi, Tokyo, Japan).

Yeast strains were identified using both morphological and molecular methods. Firstly, cells were scraped onto Wallerstein Laboratory nutrient agar and cultured for 72 h. The characteristics of the colony and cellular morphology were examined and photographed with a microscope (Olympus, Tokyo, Japan). Next, genomic DNA was extracted from three ethanol-tolerant strains (C6, F112, and F15) using a DNA extraction kit (B518257; Sangon Biotech, China) following the manufacturer’s instructions. The D1/D2 domain within the 26S rDNA was amplified using the polymerase chain reaction (PCR). The yeast species were then determined by comparing the 26S rDNA D1/D2 domain sequences in the GenBank database.

### Growth curve detection and sugar metabolism analysis of ethanol-tolerant non-*Saccharomyces* yeast strains

The C6, F112, and F15 strains were inoculated into YEPD broth at a concentration of 10^8^ cfu/ml and cultured under agitation at 180 rpm and 28°C for 48 h. The OD of the cultures was measured at the wavelength of 600 nm every 4 h, and a growth curve was plotted based on the time and OD_600__nm_ values.

Strains C6, F112, and F15 were inoculated with a concentration of 10^8^ cfu/ml into a 0.6% yeast powder solution containing 2% final concentration of glucose, sucrose, maltose, lactose, and galactose, respectively. The yeast powder solution was placed in test tubes containing Durham tubes and incubated at 28°C for 48 h. The formation of gas bubbles in the Durham tubes was observed. A positive reaction was recorded as “+” if bubbles formed. Otherwise, a negative reaction was recorded as “−.”

### Analysis of winemaking tolerances of ethanol-tolerant non-*Saccharomyces* yeast strains

Strains of C6, F112, and F15 were inoculated in YEPD broth at a concentration of 10^8^ cfu/ml with (1) glucose concentrations of 100, 150, 200, 250, or 300 g/L; (2) citric acid mass fractions of 1, 1.5, 2, 2.5, or 3% (w%); and (3) a sulfur dioxide contents of 50, 100, 150, 200, or 300 mg/L. All groups were cultured at 28°C and 180 rpm for 36 h with three replicates, and then OD_600__nm_ values were measured.

### Production capacity of hydrogen sulfide and β-glucosidase activity in ethanol-tolerant non-*Saccharomyces* yeast strains

The hydrogen sulfide (H_2_S) production activities of C6, F112, and F15 were investigated using BiGGY agar by comparing the depth of colony color (Caridi et al., 2022).

The ability of the strains to produce β-glucosidase was analyzed using the p-nitrophenyl-β-D-glucopyranoside (p-NPG) method. Strains C6, F112, and F15 were inoculated into YEPD medium and shaken at 180 rpm at 28°C for 72 h. The supernatant was obtained after centrifugation at 3,000 *g* for 10 min and used for the determination of enzyme activity. Enzyme activity units (U) were defined as the amount of enzyme required to produce 1 μmol of p-nitrophenol (p-NP) by hydrolyzing 1 μmol of p-NPG under conditions of pH 5.0 and 50°C for 1 min.

### Laboratory-scale fermentation of *R. roxburghii* fruit wine

Fresh, mature, and non-rotten *R. roxburghii* (Supplementary Figure 1) was juiced with juice extractor (Midea, WJE2802D, China) and then treated with 100 mg/L of potassium metabisulfite and 20 mg/L of pectinase at room temperature for 12 h. The juice was then adjusted to 24°Brix with crystalline sucrose and divided into four groups, with each group replicated in triplicate in 2 L sterile triangular flasks. For the C6 + *S.* cerevisiae X16 group, F112 + *S. cerevisiae* X16 group, and F15 + *S. cerevisiae* X16 group, each group was inoculated with 10^8^ cfu/ml of the C6, F112, or F15 strain and 10^7^ cfu/ml of *S. cerevisiae* X16, with a control group that was only inoculated with 10^7^ cfu/ml of *S. cerevisiae* X16. The flasks were left to ferment statically at 26°C until fermentation was completed. After fermentation, the *R. roxburghii* fruit wine from each group was centrifuged at 4,000 rpm for 10 min, and the supernatant was used for the determination of the quality indicators of *R. roxburghii* fruit wine.

### Analysis of flavor and quality characteristics of *R. roxburghii* fruit wine

The alcohol content, total sugar, total acidity, and volatile acid content of the *R. roxburghii* wine were determined following the methods described by Liu et al. (2021b). The sensory characteristics of th*e R. roxburghii* fruit wine were analyzed using an electronic tongue system. For this, 80 ml of each group of *R. roxburghii* fruit wine was taken and added to a dedicated beaker for the electronic tongue system. The electronic tongue system was used according to the instructions in the user manual to test each group of *R. roxburghii* fruit wine. The sampling time was 120 s, the sampling speed was 1/s, each sample was measured in triplicate, and each replicate was collected four times.

The headspace solid-phase microextraction-gas chromatography-mass spectrometry system (TQ8040, Agilent, USA) was used to analyze the aroma characteristics of the *R. roxburghii* fruit wine. The aroma components of *R. roxburghii* fruit wine were extracted at 40°C for 30 min, with cyclohexanone used as the internal standard for determining the aroma components of *R. roxburghii* fruit wine. The odor activity value (OAV) of each aroma component was calculated by referring to the threshold values of each volatile aroma component.

### Statistical analysis

Data results were presented as mean ± SD. Principal component analysis (PCA) and one-way ANOVA were performed using SPSS 21.0 to test for significant differences among the groups. A *p*-value of less than 0.05 was considered statistically significant. Each experiment was repeated in triplicate.

## Results

### Screening of ethanol-tolerant non-*Saccharomyces* yeast strains

When the native non-*Saccharomyces* yeasts isolated from *R. roxburghii* were treated with 12% (v/v) ethanol, most of them died. However, three yeast strains (designated as C6, F112, and F15) exhibited satisfactory growth with OD_600nm_ values of 0.56 ± 0.02, 0.47 ± 0.01, and 0.48 ± 0.01, respectively. Therefore, C6, F112, and F15 were selected as ethanol-tolerant strains for further analysis.

### Identification of ethanol-tolerant non-*Saccharomyces* yeast strains

**FIGURE 1:**
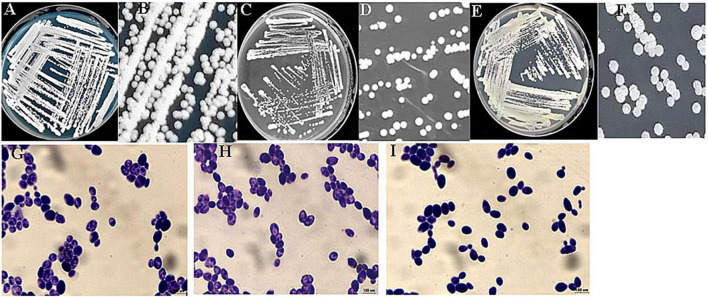
Colony and cell morphologies of ethanol-tolerant non-*Saccharomyces* yeasts isolated from *R. Roxburghii*. **(A,B)** Colony morphology of C6 on WL medium; **(C,D)** colony morphology of F112 on WL medium; **(E,F)** colony morphology of F15 on WL medium; **(G)** cell morphology of C6 following crystal violet staining (100×); **(H)** cell morphology of F112 following crystal violet staining (100×); and **(I)** cell morphology of F15 following crystal violet staining (100×).

The identification of the three ethanol-tolerant yeast strains was initially based on morphological characteristics on WL agar. As shown in Figure 1, the colony color of C6, F112, and F15 was white, and their colony topography was convex and opaque.

To confirm the identity of the ethanol-tolerant yeast strains, their 26S rDNA D1/D2 domain sequences were compared. The analysis revealed that the 26S rDNA sequences of C6, F112, and F15 had the highest similarity to *C. tropicalis*, *Pichia guilliermondii*, and *W. anomalus*, respectively. Therefore, these three strains of ethanol-tolerant yeasts (C6, F112, and F15) were identified and named *C. tropicalis* C6, *P. guilliermondii* F112, and *W. anomalus* F15 based on the results of morphological characteristics and sequence alignment.

### Growth characteristics of ethanol-tolerant non-*Saccharomyces* yeast strains

**FIGURE 2:**
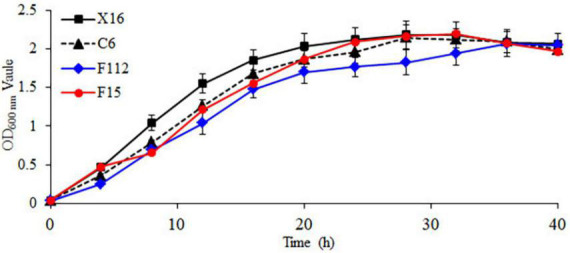
Growth curve of ethanol-tolerant non-*Saccharomyces* yeasts isolated from *R. roxburghii*.

The growth curves of the strains are shown in Figure 2, with a lag phase from 0 to 4 h, a logarithmic growth phase from 4 to 20 h, and a stationary phase after 20 h. During the logarithmic growth phase, the OD_600__nm_ values of *C. tropicalis* C6, *P. guilliermondii* F112, and *W. anomalus* F15 were all lower than those of the commercial *S. cerevisiae* X16. During the stationary phase, the OD_600__nm_ of *P. guilliermondii* F112 was lower than that of *S. cerevisiae* X16. Throughout the entire growth period, the growth of *P. guilliermondii* F112 was lower than that of *S. cerevisiae* X16, while the growth of *C. tropicalis* C6 and *W. anomalus* F15 was basically consistent with that of *S. cerevisiae* X16 in the later stages of the stationary phase.

### Winemaking condition tolerances of ethanol-tolerant non-*Saccharomyces* yeast strains

**FIGURE 3:**
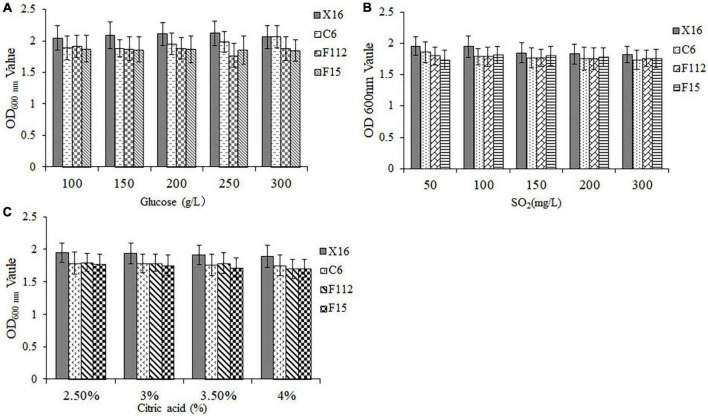
Winemaking condition tolerances of ethanol-tolerant non-*Saccharomyces* yeasts isolated from *R. roxburghii*. **(A)** Glucose tolerance; **(B)** SO_2_ tolerance; and **(C)** citric acid tolerance.

To assess the tolerance of the selected yeasts to winemaking conditions, their OD_600__nm_ values were measured after exposure to different concentrations of glucose, SO_2_, and citric acid. Results demonstrated that all three ethanol-tolerant yeast strains exhibited excellent sugar tolerance, as they were able to grow in all glucose concentrations tested (100–300 mg/L) (Figure 3A). Furthermore, *C. tropicalis* C6, *P. guilliermondii* F112, and *W. anomalus* F15 displayed similar sulfur dioxide and acid tolerance to *S. cerevisiae* X16 within the tested ranges of sulfur dioxide (50–300 mg/L) and citric acid (2.5–4.0%) concentrations, respectively (Figures 3B, C). Therefore, *C. tropicalis* C6, *P. guilliermondii* F112, and *W. anomalus* F15 showed perfect tolerance to the winemaking environment.

### Sugar metabolic performance of ethanol-tolerant non-*Saccharomyces* yeast strains

As shown in Table 1, different strains have different utilization characteristics for different sugars. *W. anomalus* F15 can only metabolize glucose with the least number of sugars that it can utilize. On the other hand, *C. tropicalis* C6 can metabolize all types of sugars except for galactose, with the broadest range of sugar utilization. *P. guilliermondii* F112 can utilize three types of sugars (glucose, sucrose, and maltose). Therefore, *C. tropicalis* C6 has the widest range of sugar metabolism, and its sugar utilization characteristics are similar to those of *S. cerevisiae* X16, except for galactose.

**TABLE 1:** Sugar utilization characteristics of ethanol-tolerant non-*Saccharomyces* yeasts isolated from *R. Roxburghii*.

Strains	Glucose	Sucrose	Maltose	Lactose	Galactose
*S. cerevisiae* X16	+	+	+	+	+
*C. tropicalis* C6	+	+	+	+	−
*P. guilliermondii* F112	+	+	+	−	−
*W. anomalus* F15	+	−	−	−	−

### Sulphureted hydrogen and β-glucosidase production abilities of ethanol-tolerant non-*Saccharomyces* yeasts strains

**FIGURE 4:**
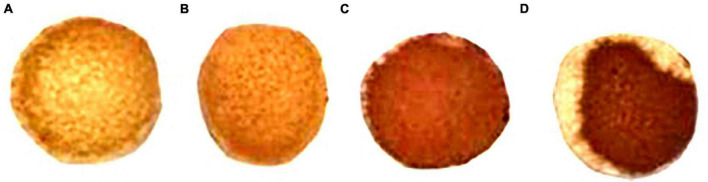
Hydrogen sulfide production ability of C6, F112, and F15 strains on BiGGY agar. **(A)** *S. cerevisiae* X16; **(B)** strain of *C. tropicalis* C6; **(C)** strain of *P. guilliermondii* F112; and **(D)** strain of *W. anomalus* F15.

The ability of ethanol-tolerant yeasts to produce H_2_S production ability was evaluated by observing the color depth on BiGGY agar (Caridi et al., 2022). As shown in Figure 4, *C. tropicalis* C6 had a similar H_2_S production ability to the control (*S. cerevisiae* X16), while *P. guilliermondii* F112 and *W. anomalus* F15 exhibited stronger H_2_S production abilities than the control, as evidenced by the colony depth color on the filter paper.

Flavor compounds in fruit are often present in the form of glycoconjugate, making them flavorless (Gueguen et al., 1996). β-Glucosidase are enzymes that could hydrolyze these glycosyl bonds, thereby releasing the flavor compounds to wines (Haslbeck et al., 2017). To investigate the β-glucosidases of the selected strains, namely, *C. tropicalis* C6, *P. guilliermondii* F112, and *W. anomalus* F15, p-NPG colorimetry was used. The result showed that the β-glucosidase production abilities of *C. tropicalis* C6 and *P. guilliermondii* F112 were similar to those of *S. cerevisiae* X16. However, the strain of *W. anomalus* F15 exhibited significantly lower β-glucosidase production ability than *S. cerevisiae* X16 (Table 2).

**TABLE 2:** β-Glucosidase production capacity of ethanol-tolerant non-*Saccharomyces* yeasts isolated from *R. Roxburghii*.

Strains	β-Glucosidase activities (U/L)
*S. cerevisiae* X16	25.6 ± 2.13a
*C. tropicalis* C6	23.5 ± 1.56a
*P. guilliermondii* F112	21.5 ± 1.87a
*W. anomalus* F15	6.3 ± 0.46b

Different lowercase letters indicate a significant difference (*P* < 0.05).

### Winemaking properties of the ethanol-tolerant non-*Saccharomyces* yeasts in laboratory-scale

The combination of *non*-*saccharomyces* yeasts with *S. cerevisiae* as fermentation starters has been widely studied and accepted in wine production (Comitini et al., 2011). To further analyze the fermentative properties of the ethanol-tolerant yeast strains, *R. roxburghii* wine was fermented by co-inoculating the *C. tropicalis* C6, *P. guilliermondii* F112, or *W. anomalus* F15 together with *S. cerevisiae* X16. Dynamic changes of the non-*Saccharomyces* yeasts population during *R. roxburghii* wine fermentation were monitored by colony counting method, and the results showed that the proportion of *C. tropicalis* C6, *P. guilliermondii* F112, and *W. anomalus* F15 gradually decreased, in contrast, the proportion of *S. cerevisiae* X16 gradually increased and dominate at the middle and later periods of fermentation (Supplementary Figure 2).

The physicochemical parameters of the fermented *R. roxburghii* wines are listed in Table 3. Ethanol degrees of wines fermented by *P. guilliermondii* F112 or *W. anomalus* F15 were lower than the wine produced by *S. cerevisiae* X16 alone, while the *C. tropicalis* C6 fermented wine was similar to that produced by *S. cerevisiae* X16. The pH and volatile acidity parameters of the four groups of *R. roxburghii* wines were similar, with no differences found among them. The total acidity were lower in the *C. tropicalis* C6 and *P. guilliermondii* F112 groups compared to the *S. cerevisiae* X16 group.

**TABLE 3:** Oenological parameters of *R. roxburghii* wine fermented with ethanol-tolerant non-*Saccharomyces* yeasts in combination with *S. cerevisiae*.

Strains	Ethanol (% v/v)	pH	Total acidity (g/L)	Volatile acidity (g/L)
*S. cerevisiae* X16	13.79 ± 0.17a	3.71 ± 0.05a	14.06 ± 0.27a	0.26 ± 0.01a
*C. tropicalis* C6 + *S. cerevisiae* X16	13.34 ± 0.11a	3.77 ± 0.03a	13.34 ± 0.32b	0.22 ± 0.00a
*P. guilliermondii* F112 + *S. cerevisiae* X16	11.51 ± 0.26b	3.76 ± 0.03a	10.92 ± 0.32c	0.22 ± 0.00a
*W. anomalus* F15 + *S. cerevisiae* X16	9.28 ± 0.68c	3.76 ± 0.05a	14.37 ± 0.11a	0.28 ± 0.01a

Different lowercase letters indicate a significant difference (*P* < 0.05).

**FIGURE 5:**
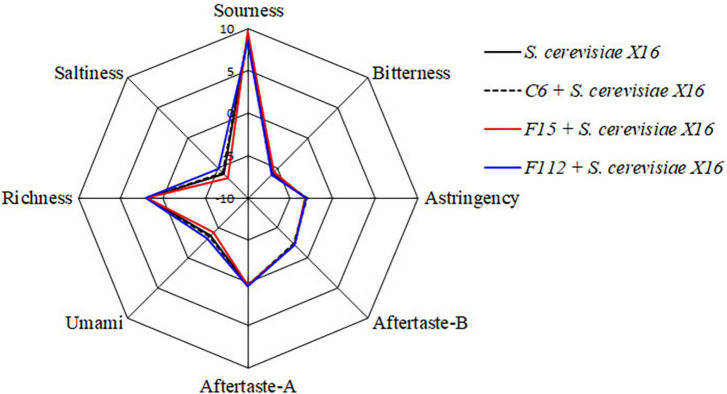
Radar chart of taste attribute of *R. Roxburghii* wine fermented with ethanol-tolerant non-*Saccharomyces* yeasts in combination with *S. cerevisiae*.

In addition, an electronic tongue system was used to perform sensory analysis and differentiate the sensory characteristics of the *R. roxburghii* wines fermented with different yeast strains. However, no significant differences in the sensory characteristics including in sourness, bitterness, astringency, aftertaste-A, aftertaste-B, umami, richness, and saltiness were found among the three types of *R. roxburghii* wines (Figure 5).

The volatile aroma profiles of the *R. roxburghii* wines fermented with the selected yeast strains were further examined by using GC-MS analysis. A total of 66 volatile compounds, including 32 volatile esters, 10 volatile alcohols, 6 volatile acids, 3 volatile aldoketones, and 15 other volatile chemicals, were identified in the four groups of fermented *R. roxburghii* wines (Table 4). The *R. roxburghii* wines co-fermented with the three ethanol-tolerant yeast strains contained 50, 46, and 55 volatile compounds, respectively, whereas only 44 volatile compounds were detected in the *R. roxburghii* wine inoculated with *S. cerevisiae* X16 alone. Additionally, 22 chemicals, including 10 esters, 1 alcohol, 1 acid, and 10 other compounds, were specifically detected in the *R. roxburghii* wines inoculated with the ethanol-tolerant yeasts. On the other hand, octyl acetate, decanoic acid, 1-nonanal, and 2,4-di-tert-butylphenol were specifically discovered in the *S. cerevisiae* X16 group. Overall, the co-inoculation of these ethanol-tolerant yeast strains isolated from *R. roxburghii* along with *S. cerevisiae* increased the types of volatile compounds in the wine (Supplementary Figure 3).

**TABLE 4:** Volatile compounds (mg/L) in *R. roxburghii* wines fermented with ethanol-tolerant non-*Saccharomyces* yeasts combined with *S. cerevisiae*.

Number	Volatile compound	*S. cerevisiae* X16	*C. tropicalis* C6 + *S. cerevisiae* X16	*P. guilliermondii* F112 + *S. cerevisiae* X16	*W. anomalus* F15 + *S. cerevisiae* X16
1	Ethyl acetate	60.80 ± 2.14a	61.87 ± 2.05a	57.57 ± 2.35a	61.70 ± 1.89a
2	Ethyl butyrate	5.77 ± 0.32a	4.46 ± 0.31b	4.07 ± 0.25b	4.43 ± 0.34b
3	Ethyl hexanoate	92.34 ± 4.65b	113.22 ± 5.87a	92.66 ± 5.19b	106.08 ± 6.11a
4	Ethyl 3-hexenoate	13.38 ± 0.57a	13.66 ± 0.48a	11.98 ± 0.52b	13.19 ± 0.44a
5	Ethyl octanoate	351.52 ± 26.51c	772.47 ± 48.79a	590.43 ± 37.49b	702.99 ± 49.68a
6	Ethyl pelargonate	8.71 ± 0.64a	3.63 ± 0.28b	3.37 ± 0.32b	3.44 ± 0.29b
7	Ethyl caprate	341.86 ± 30.51c	676.25 ± 46.18a	521.90 ± 40.62b	604.28 ± 49.53a
8	Ethyl 9-decenoate	51.71 ± 3.68a	6.53 ± 0.55b	4.97 ± 0.30c	5.78 ± 0.39b
9	Ethyl laurate	68.89 ± 3.54b	99.12 ± 6.98a	92.09 ± 7.04a	103.17 ± 8.79a
10	Ethyl isobutyrate	11.12 ± 0.43a	1.44 ± 0.09c	/	1.78 ± 0.07b
11	Ethyl 2-methyl butyrate	7.35 ± 0.38a	0.59 ± 0.02b	0.57 ± 0.01b	0.57 ± 0.02b
12	Ethyl tetradecanoate	/	10.45 ± 0.97b	15.64 ± 0.64a	14.16 ± 1.12a
13	Ethyl cinnamate	/	4.63 ± 0.31a	4.56 ± 0.29a	/
14	Ethyl palmitate	/	8.05 ± 0.35	/	/
15	Ethyl isovalerate	/	/	/	0.52 ± 0.06
16	Ethyl phenylacetate	/	/	1.97 ± 0.16a	2.14 ± 0.20a
17	Ethyl pentadecanoate	/	1.73 ± 0.21b	2.81 ± 0.27a	/
18	Ethyl benzoate	/	/	1.91 ± 0.13	/
19	Hexyl acetate	23.24 ± 0.87b	32.55 ± 1.15a	24.04 ± 1.09b	30.78 ± 1.64a
20	(E)-3-hexene-1-ol acetate	4.81 ± 0.18c	11.73 ± 0.87b	17.84 ± 1.64a	1.50 ± 0.09d
21	Isobutyl acetate	35.32 ± 2.64a	4.53 ± 0.25b	4.53 ± 0.31b	4.81 ± 0.19b
22	Isoamyl acetate	210.13 ± 13.15b	352.63 ± 17.22a	305.03 ± 16.18a	356.55 ± 20.87a
23	Isoamyl caprylate	12.74 ± 0.95b	16.69 ± 1.26a	14.04 ± 1.07a	16.29 ± 1.32a
24	Isoamyl decanoate	3.58 ± 0.16b	12.79 ± 0.59a	11.50 ± 0.62a	13.35 ± 0.79a
25	Isobutyl caprylate	15.68 ± 1.26a	4.48 ± 0.31b	/	4.57 ± 0.38b
26	Isobornyl acetate	/	/	1.88 ± 0.21	/
27	Methyl octanoate	5.33 ± 0.29a	2.28 ± 0.17b	1.78 ± 0.13c	
28	Methyl caprate	34.87 ± 2.89a	4.96 ± 0.27b	3.68 ± 0.29c	
29	Octyl acetate	5.76 ± 0.35	/	/	/
30	Phenethyl acetate	36.76 ± 2.18b	68.46 ± 4.61a	69.55 ± 3.98a	74.13 ± 5.14a
31	3-Acetoxy butane-2-yl acetate	/	2.58 ± 0.26a	1.50 ± 0.18b	2.50 ± 0.13a
32	Vinyl formate	/	/	0.56 ± 0.06	/
	Σ Esters	1,401.67 ± 98.29b	2,291.78 ± 139.85a	1,862.43 ± 121.34a	2,134.44 ± 149.76a
33	2,3-Butanediol	4.89 ± 0.35b	7.40 ± 0.62a	4.90 ± 0.29b	6.18 ± 0.61a
34	3-Hexene-1-ol	17.17 ± 1.63c	56.07 ± 4.36a	60.00 ± 4.62a	48.05 ± 3.75b
35	Hexanol	8.65 ± 0.62b	14.64 ± 0.98a	16.51 ± 0.89a	15.61 ± 1.26a
36	Isobutanol	26.01 ± 1.89c	47.03 ± 3.25b	62.79 ± 4.62a	53.56 ± 3.69a
37	Isoamylol	459.51 ± 29.45b	759.99 ± 52.92a	847.94 ± 65.68a	800.96 ± 59.78a
38	3-Methylthiopropanol	/	2.44 ± 0.20a	2.48 ± 0.29a	2.53 ± 0.18a
39	Methanol	3.29 ± 0.26a	3.57 ± 0.32a	1.91 ± 0.15b	2.07 ± 0.19b
40	Octanol	3.18 ± 0.26a	3.63 ± 0.38a	2.96 ± 0.26a	3.52 ± 0.32a
41	Phenethyl alcohol	146.31 ± 7.96b	245.58 ± 16.65a	259.68 ± 14.89a	276.89 ± 16.92a
42	Propanol	2.31 ± 0.20b	2.76 ± 0.23a	3.03 ± 0.28a	2.74 ± 0.18a
	Σ Alcohols	671.32 ± 42.62b	1,143.11 ± 79.91a	1,262.2 ± 91.97a	1,212.11 ± 86.88a
43	Acetic acid	11.47 ± 0.89c	17.76 ± 0.97b	17.05 ± 1.11b	21.12 ± 1.96a
44	n-Decanoic acid	5.98 ± 0.45	/	/	/
45	Isobutyric acid	5.82 ± 0.57a	2.74 ± 0.31c	/	3.63 ± 0.29b
46	Isovaleric acid	/	/	/	8.99 ± 0.62
47	3-Methylvaleric acid	3.08 ± 0.21b	5.74 ± 0.42a	6.00 ± 0.32a	6.56 ± 0.46a
48	Octanoic acid	14.34 ± 1.14b	19.74 ± 1.56a	19.08 ± 1.62a	20.22 ± 1.45a
	Σ Acids	40.69 ± 3.26 b	45.98 ± 3.26b	42.13 ± 3.05 b	60.52 ± 4.78 a
49	Acetaldehyde	10.84 ± 1.87a	0.44 ± 0.05b	/	0.55 ± 0.07b
50	4-Methoxy-2,5-dimethyl-3(2H)-furanone	35.39 ± 2.65a	4.77 ± 0.32b	5.31 ± 0.38b	5.32 ± 0.29b
51	1-Nonanal	4.14 ± 0.23	/	/	/
52	Σ Aldoketones	50.37 ± 4.75a	5.21 ± 0.37b	5.31 ± 0.38b	5.87 ± 0.36b
53	Benzothiazole	/	2.43 ± 0.16	/	/
54	Borane-methyl sulfide complex	/	0.60 ± 0.58a	0.68 ± 0.39a	0.74 ± 0.42a
55	1,3,5,7-Cyclooctatetraene	0.89 ± 0.19b	5.49 ± 0.36a	/	/
56	Dodecane	3.84 ± 0.36a	3.21 ± 0.29a	/	2.94 ± 0.25b
57	2,4-Di-tert-butylphenol	42.10 ± 3.27	/	/	/
58	Dipentene	36.75 ± 2.35a	5.75 ± 0.34b	4.77 ± 0.16c	5.62 ± 0.64b
59	Dimethyl sulfide	/	/	/	0.52 ± 0.02
60	n-Heptadecane	/	/	/	1.50 ± 0.10
61	2-Methyl-1,5-dioxaspiro[5.5]undecane	/	3.58 ± 0.21a	1.74 ± 0.12b	1.40 ± 0.11c
62	Naphthaline	9.89 ± 0.95b	24.95 ± 1.56a	23.35 ± 1.98a	23.99 ± 1.85a
63	α-p-Dimethylstyrene	/	/	/	4.03 ± 0.23
64	n-Pentadecane	/	/	/	2.12 ± 0.12
65	p-Cymene	/	/	/	0.55 ± 0.03
66	Styrene	/	4.64 ± 0.35a	/	4.16 ± 0.29a
63	Tetradecane	/	/	2.14 ± 0.17b	3.02 ± 0.23a
	Σ Other compounds	93.07 ± 7.12a	50.65 ± 3.85b	32.68 ± 2.82c	50.59 ± 4.29b

The symbol “/” represents a compound that is not detected. Different lowercase letters indicate a significant difference (*P* < 0.05).

When checking the volatile compound contents of *R. roxburghii* wines with the different ethanol-tolerant yeast strains, variations in the levels of volatile esters, alcohols, acids, aldoketones, and other chemicals were observed (Table 4). Co-inoculation with the three ethanol-tolerant yeasts resulted in an increase in volatile esters and alcohols, as well as a decrease in volatile aldoketones and other compounds (Table 4). In addition, *R. roxburghii* wines co-fermented with *W. anomalus* F15 and *S. cerevisiae* X16 exhibited higher levels of volatile acids compared to those produced with *S. cerevisiae* X16 alone.

The OAV was used to further evaluate the contribution of the main aromatic compounds to the aromatic characteristics of *R. roxburghii* wine. Compounds with OAV greater than 1 were considered to have a significant impact on the aroma, while those with OAV less than 1 were considered less important. Table 5 shows the calculated OAVs of twenty aromatic compounds in *R. roxburghii* wine. Thirteen compounds had OAVs greater than 1, while only 1 had an OAV less than 1 across all 4 groups of *R. roxburghii* wine. Specifically, ethyl 9-decenoate had an OAV greater than 1 only in the *S. cerevisiae* X16 group, whereas isobutanol had the only OAV less than 1 in the same group. The OAVs of ethyl caprate, isoamyl acetate, ethyl hexanoate, and ethyl octanoate were high in all four groups, suggesting that these compounds strongly contribute to the aroma of *R. roxburghii* wine.

**TABLE 5:** The OAVs for the main compounds in *R. roxburghii* wine fermented with different yeasts.

Number	Volatile compound	Odor descriptor	Odor threshold (mg/L)	OAV			
				***S. cerevisiae* X16**	***C. tropicalis* C6 + *S. cerevisiae* X16**	***P. guilliermondii* F112 + *S. cerevisiae* X16**	***W. anomalus* F15 + *S. cerevisiae* X16**
A1	Ethyl acetate	Pineapple, fruity, solvent, balsamic	0.75	81.07	82.49	76.77	82.27
A2	Ethyl butyrate	Fruity	0.02	288.5	223	203.5	221.5
A3	Ethyl hexanoate		0.05	1,846.8	2,264.4	1,853.2	2,121.6
A4	Ethyl octanoate	Sweet, fruity	0.58	606.07	1,331.84	1,017.98	1,212.05
A5	Ethyl caprate	Sweet, fruity	0.20	1,709.3	3,381.25	2,609.5	3,021.25
A6	Ethyl 9-decenoate	Roses	14.10	3.67	0.46	0.35	0.41
A7	Ethyl laurate	Fruity, fatty	0.64	107.64	154.88	143.89	161.20
A8	Ethyl cinnamate	Fruity	0.01	/	463	456	/
A9	Ethyl palmitate		1.5	/	5.37	/	/
A10	Isobutyl acetate	Sweet, fruity, apple, banana	1.6	22.08	2.83	2.83	3.01
A11	Isoamyl acetate	Banana, fruity, sweet	0.16	1,313.31	2,203.93	1,906.44	2,228.44
A12	Phenethyl acetate	Floral	1.8	20.42	38.03	38.63	41.18
B1	2,3-Butanediol	Fruity	150	0.03	0.05	0.03	0.04
B2	Hexanol	Flower, green, cut grass	8	1.08	1.83	2.06	1.95
B3	Isobutanol		40	0.65	1.18	1.57	1.34
B4	Isoamylol	Burnt, alcohol	30	15.32	25.33	28.26	26.70
B5	Phenethyl alcohol	Floral, roses	10	14.63	24.56	25.97	27.69
C1	n-Decanoic acid	Fatty and rancid	6	1.00	/	/	/
C2	Isobutyric acid	Acid, fatty	0.23	25.30	11.91	/	15.78
C3	Octanoic acid	Fatty and rancid	10	1.43	1.97	1.91	2.02

The symbol “/” represents a compound that is not detected.

**FIGURE 6:**
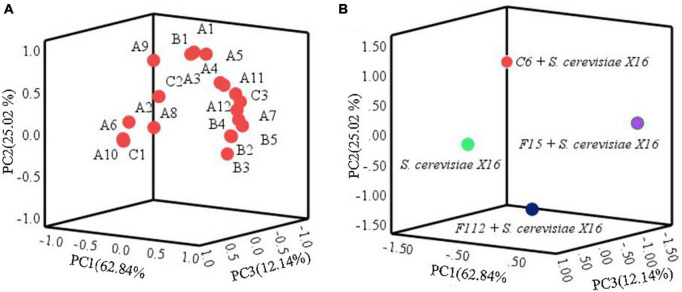
Principal component analysis of *R. roxburghii* wine fermentation with ethanol-tolerant non-*Saccharomyces* yeasts in combination with *S. cerevisiae*. **(A)** Principal component load plot of volatile aroma compounds; and **(B)** principal component score of volatile aroma compounds. A1, ethyl acetate; A2, ethyl butyrate; A3, ethyl hexanoate; A4, ethyl octanoate; A5, ethyl caprate; A6, ethyl 9-decenoate; A7, ethyl laurate; A8, ethyl cinnamate; A9, ethyl palmitate; A10, isobutyl acetate; A11, isoamyl acetate; A12, phenethyl acetate; B1, 2,3-butanediol; B2, hexanol; B3, isobutanol; B4, isoamylol; B5, phenethyl alcohol; C1, n-decanoic acid; C2, isobutyric acid; C3, octanoic acid.

Principal component analysis was used to further assess the impact of the main aromatic compounds on the characteristics of *R. roxburghii* wine. As depicted in Figure 6, the three principal components, PC1, PC2, and PC3, accounted for 62.84, 25.02, and 12.14% of the total variance, respectively, explaining 100.00% of the total variance. Most of the compounds were clustered in the positive axis of PC1 and PC2, and significant differences in distribution were observed among the four groups of fermented *R. roxburghii* wine. Ethyl palmitate might be closely related to the mixed fermentation of *C. tropicalis* C6 and *S. cerevisiae* X16, while *W. anomalus* F15 and *S. cerevisiae* X16 produced wines were characterized by compounds located in the positive PC1 and PC2, such as fruity and rosy chemicals (e.g., isobutyl acetate and ethyl 9-decenoate), which may contribute to the aroma of *S. cerevisiae* X16 fermented wine. However, it was challenging to identify the main volatile characteristics of *P. guilliermondii* F112 and *S. cerevisiae* X16 fermented *R. roxburghii* wines. Moreover, the mixed fermented wine using *W. anomalus* F15 and *S. cerevisiae* X16 was closely clustered to many esters, alcohols and acids, such as ethyl laurate, phenethyl alcohol, and octanoic acid, which may endow the wine more complex aroma characteristics.

## Discussion

In order to evaluate yeast strains for winemaking, ethanol tolerance is an essential property (Novo et al., 2014). Researchers have made numerous efforts to isolate ethanol-tolerant yeasts from various sources, including fruits and fermentation conditions, for industrial purposes (Osho, 2005; Tikka et al., 2013). In a recent study, ethanol-tolerant yeast flora was isolated, identified, and screened from the Indian cashew apple, and seven strains of ethanol-tolerant yeasts were identified as *Candida* spp. (Desai et al., 2012). Besides, mutational breeding techniques were also applied to produce ethanol-tolerant yeasts. For example, the *Pichia terricola* H5 strain, which initially displayed 8% ethanol tolerance, was subjected to ultraviolet irradiation and diethyl sulfate mutagenesis to increase its ethanol tolerance (Gao et al., 2022). As a result, two mutant strains (UV5 and UV8) that demonstrated high tolerance to ethanol were obtained, and modified aroma profiles were evident in the fermentation samples exposed to these strains. In the present study, ethanol-tolerant non-*Saccharomyces* yeasts were screened for the first time from *R. roxburghii*. Three strains of yeasts displaying high ethanol tolerance were successfully obtained and subsequently identified as *C. tropicalis*, *P. guilliermondii*, and *W. anomalus* (Figure 1).

While *S. cerevisiae* is the most commonly isolated ethanol-tolerant yeast and widely used for wine making (Alexandre et al., 2004), non-*Saccharomyces* yeasts have been considered sensitive to ethanol and are typically dominant in the early stage of spontaneous wine fermentation (Liu et al., 2021c). However, some non-*Saccharomyces* species were proved to be tolerant to ethanol. For example, three strains of *Candida* spp. yeast isolated from Indian cashew apple were able to tolerate up to 10% (v/v) ethanol (Desai et al., 2012). Besides, a strain of *Candida stellata* was found to produce ethanol levels up to 13.48% (v/v) during the fermentation of Macabeo grape must, indicating a high tolerance to ethanol (Clemente-Jimenez et al., 2004). In this study, we obtained a strain of *C. tropicalis* that exhibited robust growth in YEPD broth containing 12% (v/v) of ethanol (Figure 1). These results indicated that *Candida* species may be a better source for screening ethanol-tolerant yeast.

Among non-*Saccharomyces* yeasts, *W. anomalus* has gained increasing attention in recent years due to its unique physiological characteristics and metabolic features (Liu et al., 2021d). These yeast species have been reported to tolerate various extreme environmental conditions such as high/low pH, high osmotic pressure, and anaerobic conditions (Schneider et al., 2012). In our previous study, a fruity aroma-producing strain of *W. anomalus* C11 was isolated from *R. roxburghii*, which was capable of withstanding 9% (v/v) ethanol treatment (Liu et al., 2021b). In the present study, we isolated another strain of *W. anomalus* F15 from *R. roxburghii*, which displayed a higher ethanol tolerance of up to 12% (v/v) than *W. anomalus* C11. Moreover, *W. anomalus* F15 was also found to be tolerant to glucose, sulfur dioxide, and citric acid, suggesting that this strain of *W. anomalus* may have a better potential for application in winemaking (Figure 3).

Aroma characteristic is an important parameter in assessing wine quality (Styger et al., 2011). Combining non-*Saccharomyces* starters with *S. cerevisiae* during winemaking to enhance the richness and complexity of wine has been widely accepted by researchers and wine producers (Contreras et al., 2015). In this study, *R. roxburghii* wines were fermented by co-inoculating ethanol-tolerant non-*Saccharomyces* yeasts with *S. cerevisiae*. The results showed that the levels of volatile esters and alcohol compounds significantly increased in the three mixed-fermentation wines compared to those fermented with *S. cerevisiae* alone. On the other hand, the levels of aldoketones and other compounds significantly decreased in mixed-fermentation wines (Table 4 and Supplementary Figure 2). Therefore, co-inoculating ethanol-tolerant non-*Saccharomyces* yeasts with *S. cerevisiae* can regulate the aromatic characteristics of *R. roxburghii* wine, contributing to the enrichment of different types of *R. roxburghii* wine.

Ester compounds are a type of metabolite generated during alcohol metabolism (Sumby et al., 2021). Most ester compounds exhibit floral and fruity aromatic characteristics and are important contributors to the aroma profiles of various fermented wines (Rojas et al., 2001). Our study found that using native non-*Saccharomyces* yeasts of *R. roxburghii* in combination with commercial *S. cerevisiae* can increase the diversity and concentration of volatile esters compounds (Table 4 and Supplementary Figure 2). For example, the levels of ethyl octanoate, ethyl caprate, and ethyl laurate in non-*Saccharomyces* yeasts-fermented wines were higher than those in *R. roxburghii* wine fermented with *S. cerevisiae* alone (Table 4). Additionally, seven types of ethyl eater chemicals, including ethyl tetradecanoate, ethyl cinnamate, and ethyl palmitate, were specifically detected in the three types of *R. roxburghii* wines produced with the native non-*Saccharomyces* yeasts of *R. roxburghii* (Table 4).

## Conclusion

This study represents the first report on the screening and oenological property analysis of ethanol-tolerant non-*Saccharomyces* yeasts isolated from *R. roxburghii*. We obtained three strains of ethanol-tolerant yeasts designated as C6, F112, and F15, which were identified as *C. tropicalis*, *P. guilliermondii*, and *W. anomalus*, respectively, after treating them with 12% (v/v) of ethanol. These strains showed similar winemaking condition tolerances to *S. cerevisiae* X16, but their growth, sugar metabolic performance, and activities of sulphureted hydrogen production were different. The β-glucosidase production ability of strain *W. anomalus* F15 was lower than that of *S. cerevisiae* X16, and strains of *C. tropicalis* C6 and *P. guilliermondii* F112 were similar to *S. cerevisiae* X16. Mixed inoculation of these ethanol-tolerant yeast strains with *S. cerevisiae* regulated the volatile aroma characteristics of the fermented *R. roxburghii* wine, enriching and enhancing its aroma flavor. Therefore, the selected ethanol-tolerant yeasts exhibit potential applications in the production of unique *R. roxburghii* wine.

## Data availability statement

The original contributions presented in this study are included in the article/Supplementary material, further inquiries can be directed to the corresponding authors.

## Author contributions

YL and XL wrote the original draft manuscript. PD, XT, WZ, and MK conducted the experiments. XL and MH conceived and designed the experiments. All authors contributed to the article and approved the submitted version.
