# Supplementary material for: Screening and oenological property analysis of ethanol-tolerant non-Saccharomyces yeasts isolated from Rosa roxburghii Tratt
Source: Front Microbiol. 2023 Jun 1;14:1202440. doi: 10.3389/fmicb.2023.1202440 (PMC10267374; doi:10.3389/fmicb.2023.1202440)

Supplementary materials

Fig.S1 External and internal morphology of *R. roxburghii* (Guinong 5) fruit.

Fig.S2 Proportion of *C. tropicalis* C6 (A), *P. guilliermondii* F112 (B), or *W. anomalus* F15 (C) during *R. roxburghii* wines fermentation.

Fig.S3 Types and amounts of aromatic compounds in *R. roxburghii* wines fermented with ethanol-tolerant non-*Saccharomyces* yeasts in combination with *S. cerevisiae*.

Types of aromatic compounds; (B) Amounts of aromatic compounds. Different lowercase letters above the standard deviation bar indicate a significant difference (P < 0.05).

Fig.S1


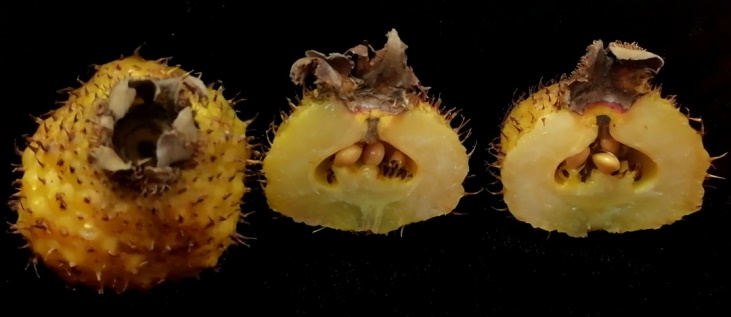


Fig.S2


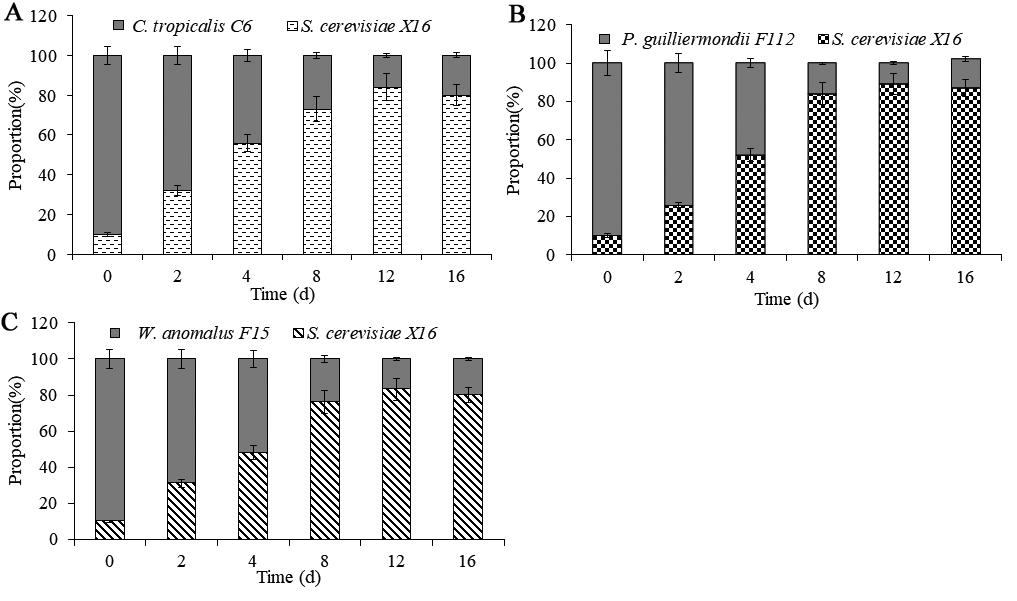


Fig.S3


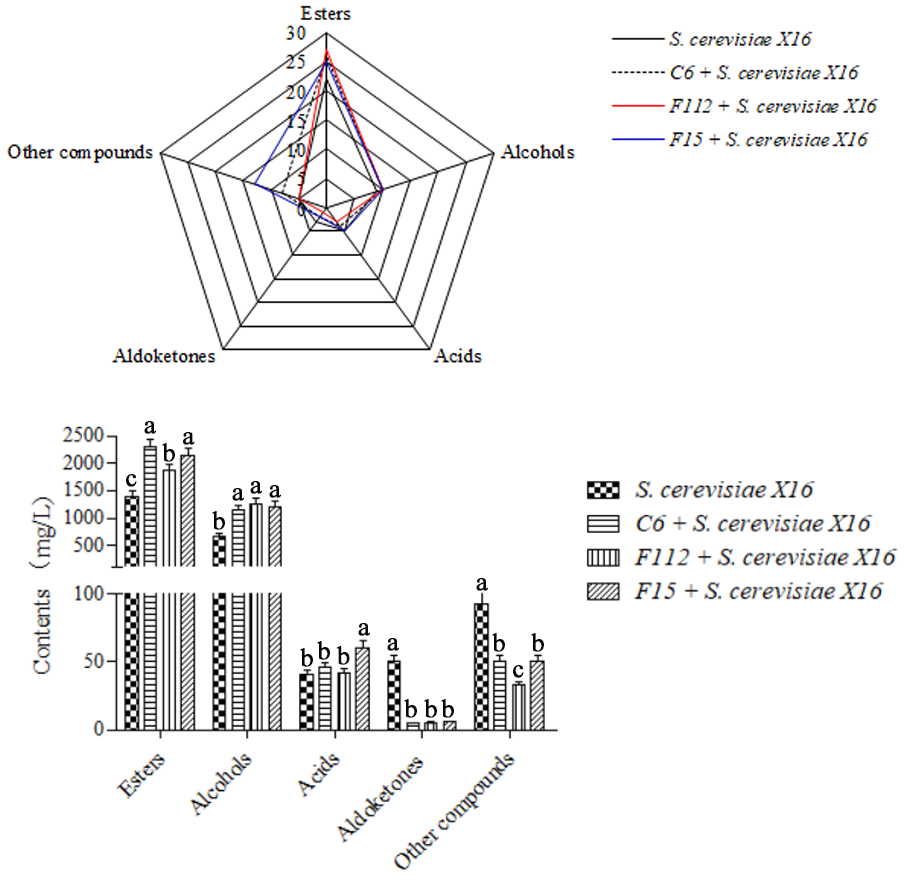

Supplement: Supplementary file 1 [file Data_Sheet_1.DOCX]
